# Unrecognized constellation of multiple congenital anomalies in a newborn: a rare case report

**DOI:** 10.1097/MS9.0000000000003150

**Published:** 2025-03-27

**Authors:** Mohanad Jaber, Usama Qumsieh, Amal M. Shawabka, Asaal I. Alwhoosh, Hiba H. AlZamareh, Bushra Kh. Pujee, Malakan Y. Hawamdeh

**Affiliations:** a Higher Specialization in Forensic Medicine. Head of ‏Clinical Medical Sciences Department,Faculty of Medicine,Palestine Polytechnic University, Hebron, Palestine; b Head of pediatric surgery department, Al-Ahli Hospital and medical center, Faculty of Medicine, Palestine Polytechnic University, Hebron, Palestine; c Faculty of Medicine,Palestine Polytechnic University, Hebron, Palestine

**Keywords:** case report, cleft lip and palate, congenital hypothyroidism, esophageal duplication cyst, multiple congenital, transverse colon atresia

## Abstract

**Introduction and importance:**

Multiple congenital anomalies occur when more than two unrelated structural anomalies are present in one case and cannot be related to a specific syndrome. Genetic, nutritional, and environmental factors play major roles in the development of such cases. Up to our knowledge and research, there are no similar cases in the literature such as this case that combine these four anomalies.

**Case presentation:**

A female neonate of preterm gestational age 34 + 1 who had cleft lip and palate was born and needed an immediate neonatal intensive care unit interface. The patient experienced several complications, including acute respiratory distress, hypocalcemia, sepsis, thrombocytopenia, and congenital hypothyroidism. The images also revealed transverse colon atresia and an esophageal duplication cyst.

**Clinical discussion:**

Management consisted of surgeries for the atresia and for the esophageal duplication cyst, thyroid replacement with hormones, and support requirements including respiratory care in many forms.

**Conclusion:**

This case highlights the necessity of a comprehensive and coordinated care plan for patients with congenital anomalies. When a newborn presents with a single defect, it is crucial for the physicians to investigate the genetic, environmental, and embryological factors that may correlate with additional anomalies. Effective management in such cases requires the seamless collaboration of multiple specialists and vigilant monitoring of multiple organ systems to ensure optimal patient outcomes.

## Introduction

Multiple congenital anomalies (MCAs) involve two or more unrelated major defects, often caused by genetic, infectious, nutritional, or environmental factors^[1]^. Preterm neonates with MCAs pose diagnostic and therapeutic challenges^[2]^. This case report describes a Palestinian newborn with four rare anomalies: esophageal duplication cysts (EDC), a benign condition causing dysphagia or respiratory distress, typically treated surgically^[3]^; congenital hypothyroidism (CH), a thyroid hormone deficiency occurring in 1 in 2000–4000 live births^[4]^; colonic atresia (CA), a rare cause of neonatal bowel obstruction, often associated with other anomalies^[5]^; and cleft lip and palate (CLP), occurring in 1 in 700 births and requiring early multidisciplinary care^[6]^. The coexistence of these anomalies suggests a shared embryological origin, as they develop embryologically during overlapping periods (6th–8th weeks)^[7]^. This highlights the importance of comprehensive evaluation in congenital anomalies, as unrelated defects may arise from early developmental disruptions.This work has been reported in line with the Surgical CAse Report 2023 guidelines^[8]^.

**HIGHLIGHTS**

- Multiple congenital anomalies described when there are more than two major structural anomalies in one patient and pose diagnostic and therapeutic dilemmas to clinicians.
- There are no reported cases that combine these four anomalies: cleft lip and palate, bowel atresia, congenital hypothyroidism, and esophageal duplication cyst.
- Esophageal duplication cyst is a very rare condition and should be suspected with a patient with recurrent lung atelectasis.
- The presence of esophageal duplication cyst, cleft lip and palate, and intestinal atresia in this patient suggests a common developmental defect during early embryological development, highlighting the importance of evaluating all congenital anomalies for concurrent defects.
- The management demanded the successful coordination of several professionals and diligent “overwatching” of several organs in the body.

## Case report

We present a case of Palestinian seven-month-old female neonate, born preterm at 34 + 1 weeks gestation on 6 April 2024, via normal delivery. She was born with a birth weight of 1900 g and had a cleft lip and palate. The newborn was therefore transferred to the neonatal intensive care unit (NICU) for further evaluation and management.

Upon admission to the NICU, the neonate exhibited respiratory distress and CO2 retention, which necessitated intubation. A chest X-ray was initially unremarkable, but she developed right lung atelectasis, which was later resolved. She remained hemodynamically stable without sedation and displayed no abnormal movements or apnea. The neonate passed urine within the first 24 hours and began feeding through an orogastric tube (OGT).

On the first day, her calcium level was low, requiring intravenous calcium administration. She later developed jitteriness that was managed with intravenous calcium gluconate. On the second day of life, the septic workup showed an increased white blood cell count of 13 × 10^9^/L and a platelet count of 191 × 10^3^. Subsequently, the baseline negative C-reactive protein (CRP) level increased to 22, and empirical antibiotic therapy consisting of ampicillin and gentamicin was administered.

**Figure 1.**
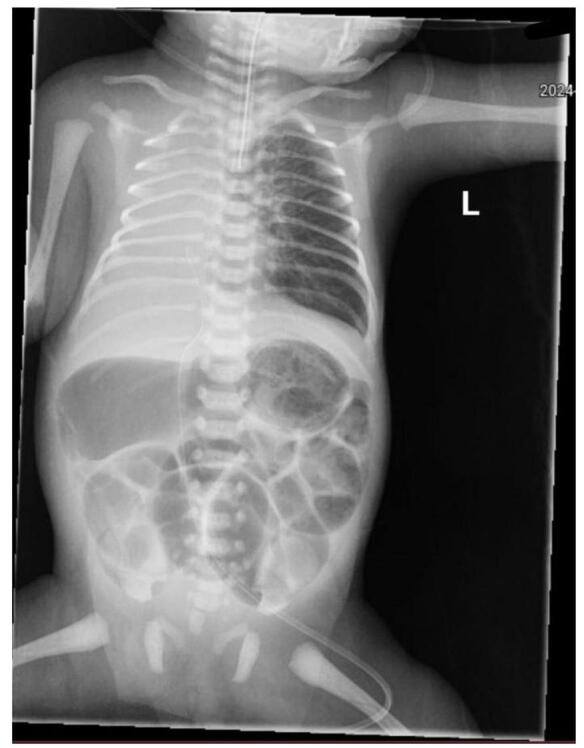
Anteroposterior (AP) plain abdominal radiograph demonstrating a disproportionately dilated loop of colon proximal to the site of atresia, with a notable transition zone and air-fluid levels, consistent with mechanical obstruction. The distal colon appears collapsed, and there is no evidence of pneumoperitoneum or abnormal calcifications.

By day three the patient had a distended, rigid abdomen and greenish material in the feeding tube without passing any meconium. An abdominal X-ray revealed dilated bowel loops and bowel atresia (Fig. 1). On 20 April 2024, exploratory laparotomy revealed transverse colon atresia, and a percutaneous endoscopic gastrostomy (PEG) tube along with a double-barrel ileostomy was performed.

On day 5, the patient developed thrombocytopenia with leukopenia, high prothrombin time (PT), and high international normalized ratio (INR). She received platelet transfusions and vitamin K. Possible lumbar puncture was maintained as thrombocytopenia risk remained a major concern. Blood culture results showed extended-spectrum beta-lactamase (ESBL)-producing Klebsiella; thus piperacillin/tazobactam and amikacin were initiated according to sensitivity tests.

Endocrinological lab tests indicated a TSH level >100 mIU/L and T4 levels <0.4 µg/dL. An ACTH stimulation test ruled out primary adrenal hypoplasia. The patient was diagnosed with primary hypothyroidism and started on levothyroxine therapy.

**Figure 2.**
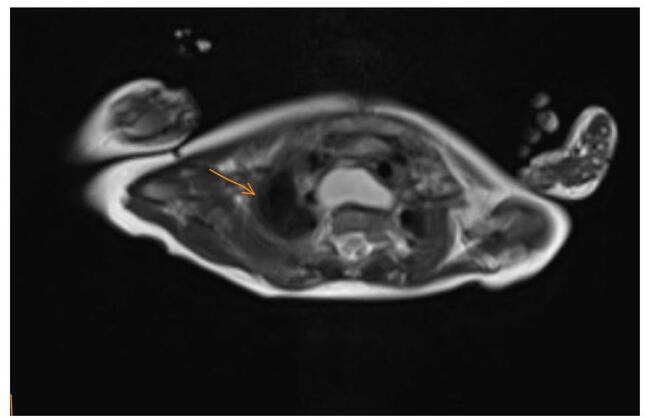
Axial chest computed tomography (CT) image demonstrating a well-defined, rounded mass in the mediastinum adjacent to the esophagus (orange arrow). The lesion exhibits homogeneous density and smooth margins. No significant compression of surrounding structures is observed. The findings were later confirmed to represent an esophageal duplication cyst upon further diagnostic evaluation.

**Figure 3.**
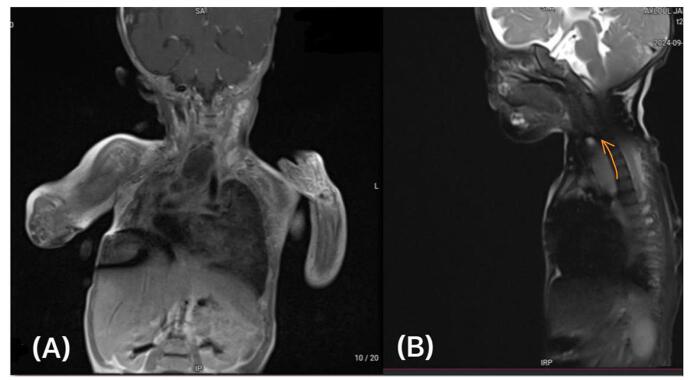
(A) Multiplanar/multisequence T1-weighted imaging of the neck and chest (without intravenous contrast) reveals a lobulated, tubular, thin-walled cystic lesion. The lesion appears hypointense on T1-weighted imaging, with no significant enhancement post-contrast. Located in the posterior mediastinum, it extends tortuously for approximately 5.4 cm from the esophageal hiatus (CS level) to the D6 level, adjacent to the anterior esophageal wall. Maximum diameters: 1.6 cm (anterior-posterior) and 1.2 cm (transverse). Findings are consistent with a large esophageal duplication cyst. (B) Lateral T1-weighted MRI of the neck shows the same large, hypointense cystic lesion (orange arrow) near the esophagus, confirming the diagnosis. The cyst has smooth margins and no signs of tissue invasion.

The patient developed recurrent atelectasis, in which chest CT was done and showed esophageal duplication cyst (Fig. 2). On 12 September 2024, neck magnetic resonance imaging (MRI) was done before the esophagectomy (Fig. 3) to confirm the diagnosis. So on September 16th, 2024, a total esophagectomy was done as the native esophagus was very narrow with septated large cysts along it. It was sent to the pathology lab, and reports were negative for dysplasia or malignancy. Post-esophagectomy, the patient started on vancomycin and meropenem as prophylaxis. Chronological Summary of Clinical Events from Birth to Last Documented Intervention is revealed in (Table 1).

**Table 1:** Chronological summary of clinical events from birth to last documented intervention

Date	Findings	Interventions
**6 April 2024 (Birth)**	Preterm delivery at 34 + 1 weeks, birth weight 1900 g. Cleft lip and palate noted.	Transferred to NICU for evaluation and management.
**6 April 2024**	Respiratory distress and CO2 retention. Right lung atelectasis (resolved). Low calcium levels.	Intubation for respiratory support. IV calcium administered. Feeding via OGT initiated.
**7 April 2024**	Jitteriness. Septic workup: WBC 13 × 10^9^/L, platelets 191 × 10^3^, CRP increased to 22.	IV calcium gluconate for jitteriness. Empirical antibiotics (ampicillin and gentamicin) initiated.
**8 April 2024**	Distended, rigid abdomen; greenish material in OGT without meconium passage. Abdominal X-ray: Dilated bowel loops, bowel atresia.	Exploratory laparotomy. Transverse colon atresia diagnosed. PEG tube and double-barrel ileostomy performed.
**9 April 2024**	Thrombocytopenia, leukopenia, elevated PT/INR. Blood culture: ESBL-producing Klebsiella.	Platelet transfusions and vitamin K administered. Antibiotics according to the sensitivty.
	Endocrinological tests: TSH >100 mIU/L, T4 < 0.4 µg/dL. ACTH stimulation test ruled out adrenal hypoplasia.	Diagnosed with primary hypothyroidism; levothyroxine therapy initiated.
**15 May 2024**	Chest CT: Esophageal duplication cyst identified.	Further diagnostic workup initiated.
**12 September 2024**	Neck MRI: Large esophageal duplication cyst confirmed.	Preoperative planning for esophagectomy.
**16 September 2024**	Narrow native esophagus with septated cysts. Pathology: Negative for dysplasia or malignancy.	Total esophagectomy performed. Postoperative prophylaxis: Vancomycin and meropenem initiated.

The family history was unremarkable for similar cases. The mother did not undergo regular prenatal care during the pregnancy, and as a result, no abnormalities were documented or identified during this period.

## Discussion

MCAs, either two or more, can be classified as syndromic according to a recognized pattern or shall be unclassified when there is no predilection for the affected organ system. They seem to be genetically complex with contributions from hereditary and environmental factors^[9]^. We present a rare case of a seven-month-old preterm baby who is currently in the NICU. She was diagnosed with several congenital malformations, including EDC, CH, CA, and CLP. After taking the mother’s medical history, which showed that there is no history of any medications, no consanguinity between the mother and father, and the prenatal detailed ultrasound did not reveal any of these abnormalities. Genetic tests were not performed due to financial constraints.

EDC is a very rare congenital embryonal malformation with an estimated prevalence of 0.0122%^[10]^. In congenital EDC, they have a double layer of surrounding smooth muscle lined by squamous or enteric epithelium and are either attached to the esophagus or located within the mediastinal wall, and they are commonly diagnosed in childhood and may remain asymptomatic to adulthood^[11]^. In our case the patient presented with recurrent atelectasis which warranted more investigations to discover the cause. There are two cases similar to ours, as the patients were diagnosed with EDC, and they presented recurrent chest infection and stridor^[12]^.

CA is one of the rarest causes of neonatal intestinal obstructions, with an reported prevalence of 1:20 000 to 1:66 000 live births. It presents with delayed meconium passage, abdominal distention, and bilious vomiting^[5]^. It can occur alone or with other congenital anomalies like Hirschsprung’s disease, malrotation, and multiple intestinal atresias. Proper and rapid diagnosis and surgical management, initially involving a double-barrel stoma followed by anastomosis after weight gain, result in a good prognosis^[13]^. There are no reported cases of transverse colon atresia combined with CLP or EDC.

CH can be isolated and diagnosed during newborn screening programs^[14]^. Or can be associated with other congenital malformations but rarely to be associated with the other three anomalies described. In this case, there is a case where CH was associated with duodenal atresia due to an intraluminal non-fenestrated diaphragm^[15]^. It may be present with cleft lip with a mutation in FOXE1 that causes Bamforth-Lazarus syndrome, which consists of a syndrome of thyroid dysgenesis, choanal atresia, cleft palate, bifid epiglottis, and spiky hair. This syndrome cannot be ruled out as the genetic testing was not done^[16]^. Levothyroxine is the treatment of choice.The goal of treatment is to raise the T4 level and normalize TSH levels^[17]^.

CLP, occurring in 1 in 700 live births, varies by geography and race, with higher prevalence in males^[6]^. It arises from genetic and environmental factors and can be syndromic (up to 30%) or non-syndromic^[17]^. Syndromic cases are linked to congenital heart defects, craniofacial anomalies, and genetic syndromes like 22q11.2 deletion syndrome^[7]^. While CLP has been associated with gastrointestinal anomalies, its co-occurrence with transverse colon atresia or esophageal duplication cysts is exceptionally rare and previously undocumented.

The fact that the EDC, CLP, and CA were present in this patient implies that the three anomalies might have arisen from a singular developmental defect during early embryological development^[18]^. As the present knowledge suggests that all of them occur during the period of differentiation that occurs at 6–8 weeks of embryonic development as the EDC occurs between the 4th and 8th weeks of development^[19]^, the CLP occurs at 6 and 9 weeks^[20]^, and CA occurs during the 8th to 10th weeks due to the failure of the gut to recanalize^[21]^. This temporal association reinforces the need to evaluate all patients presenting with any congenital anomaly and realize that unrelated defects may have developed concurrently.

The limitations of this case report include the absence of genetic testing due to financial constraints limits identification of syndromic or genetic causes. As a single case, findings cannot be generalized, and its retrospective nature may introduce bias. The rarity of the anomaly combination (EDC, CLP, CA, and CH) and lack of comparative data restrict definitive conclusions. Future studies addressing these limitations could improve understanding and management of such complex cases.

## Data Availability

None.
